# Supplementary material for: The role of carbon catalyst coatings in the electrochemical water splitting reaction
Source: Nat Commun. 2025 May 14;16:4460. doi: 10.1038/s41467-025-59740-z (PMC12078592; doi:10.1038/s41467-025-59740-z)
Supplement: Supplementary file 1 — Supplementary Information [file 41467_2025_59740_MOESM1_ESM.pdf]

Supplementary Information

The Role of Carbon Catalyst Coatings in the Electrochemical Water Splitting Reaction

William J. V. Townsend, Diego López-Alcalá, Matthew Bird, Jack W. Jordan, Graham A. Rance, Johannes Biskupek, Ute Kaiser, José J. Baldoví, Darren Walsh, Lee Johnson, Andrei N. Khlobystov, Graham N. Newton

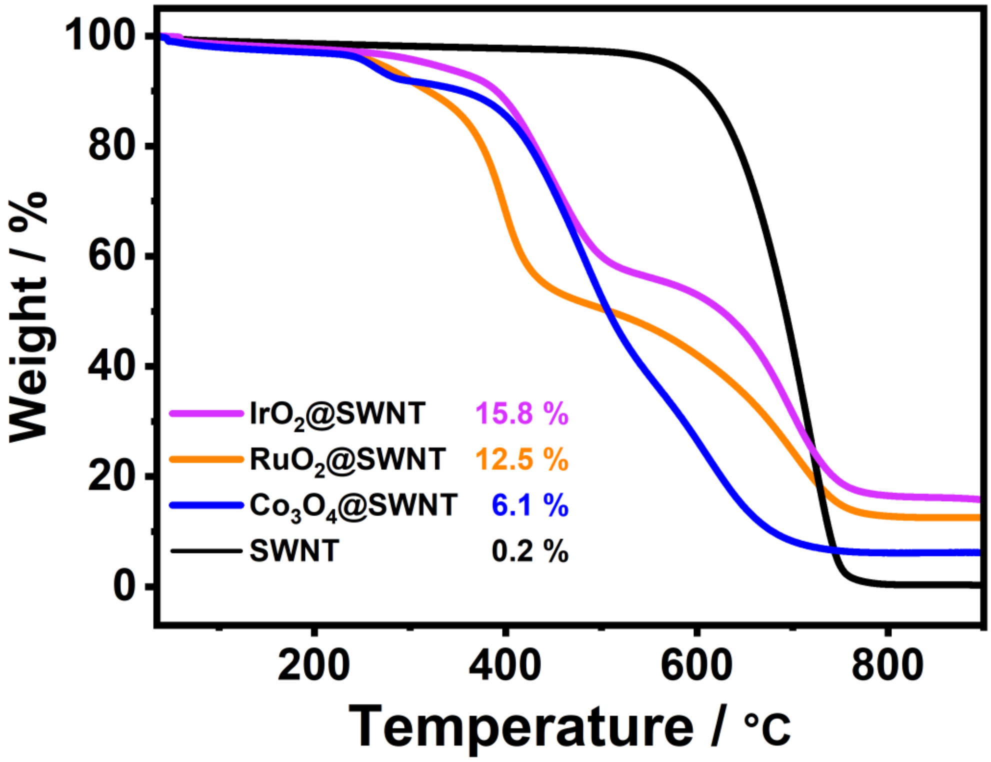

Figure S1 | Thermogravimetric analysis (TGA) traces of all catalysts. The final weight loadings are given as a weight %.

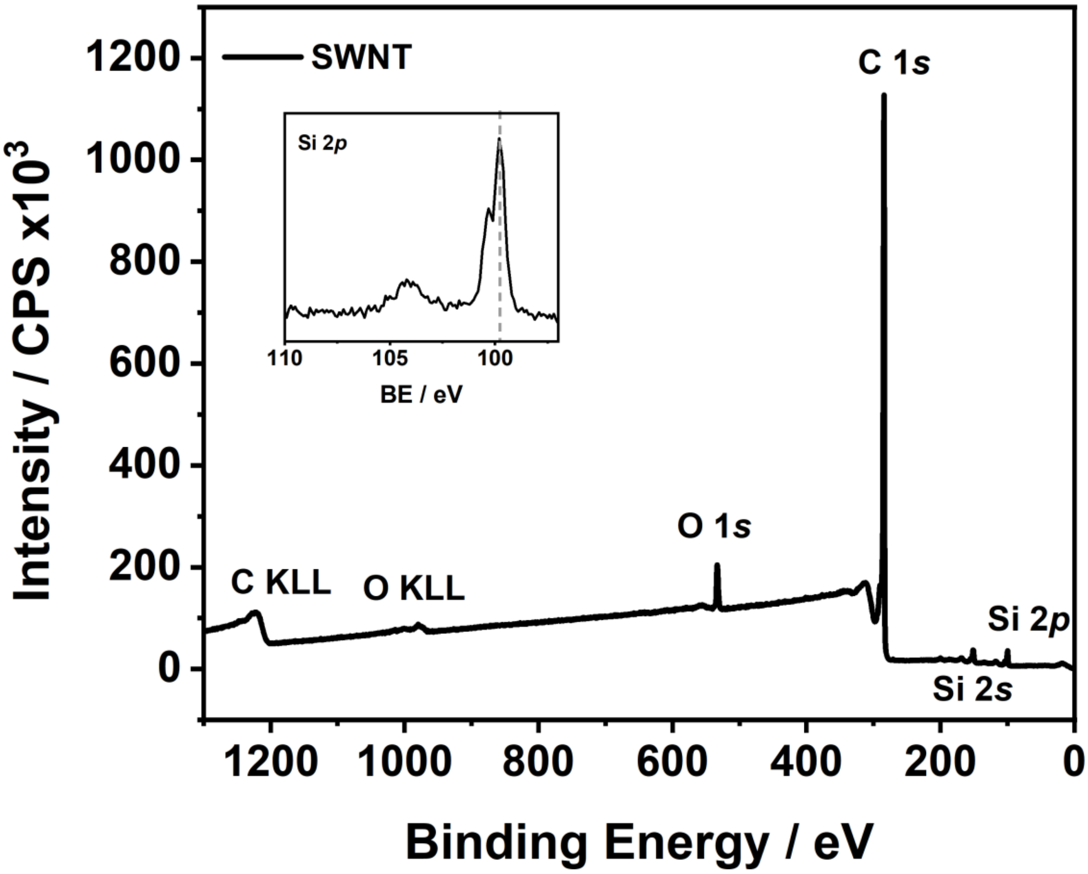

Figure S2 | XP spectrum of opened and washed SWNTs. All XP spectra shown have been reference to the silicon 2p peak, shown in inset (discussed in supplementary note X). A very small feature at 200 eV is due to a small amount of chlorine left over from HCl washing procedure.

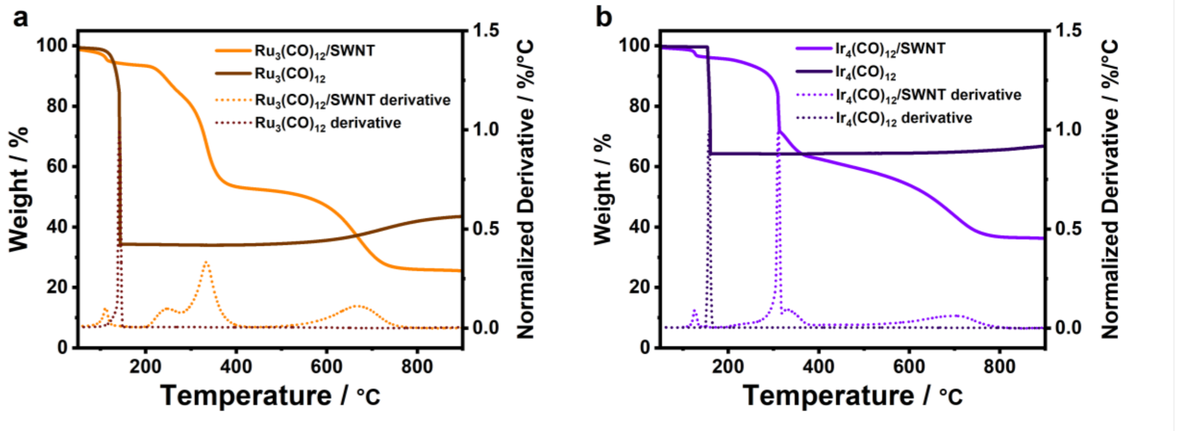

Figure S3 | Thermogravimetric analysis (TGA) traces of a) $Ru_3(CO)_{12}$ and $Ru_3(CO)_{12}$/SWNT and b) $Ir_4(CO)_{12}$ and $Ir_4(CO)_{12}$/SWNT. The derivative of each TGA trace is represented by dotted lines. The steep feature between 100 and 200 °C is due to carbonyl decomposition resulting in formation of metal oxide.

Table S1| Mass loadings, taken from TGA (Fig. S1), and defect densities taken from 532 nm Raman spectra, of the encapsulated materials and SWNT control before and after 2000 OER cycles. The defect density of each material is calculated using the integrated Raman D/G ratios.[11] Atomic percent is calculated using mass loading taken from TGA.

Catalyst	Mass loading MO_x / wt. %	Atomic percent of M / %	Defect density / μm^{-1}	2000 cycle defect density / μm^{-1}
**IrO_2@SWNT**	15.8	0.99	8.4 ± 0.2	11.8 ± 0.3
**RuO_2@SWNT**	12.5	1.27	7.5 ± 0.2	10.8 ± 0.3
**Co_3O_4@SWNT**	6.1	0.96	11.2 ± 0.3	19.3 ± 0.5
**SWNT**	N/A	N/A	5.0 ± 0.1	14.3 ± 0.4

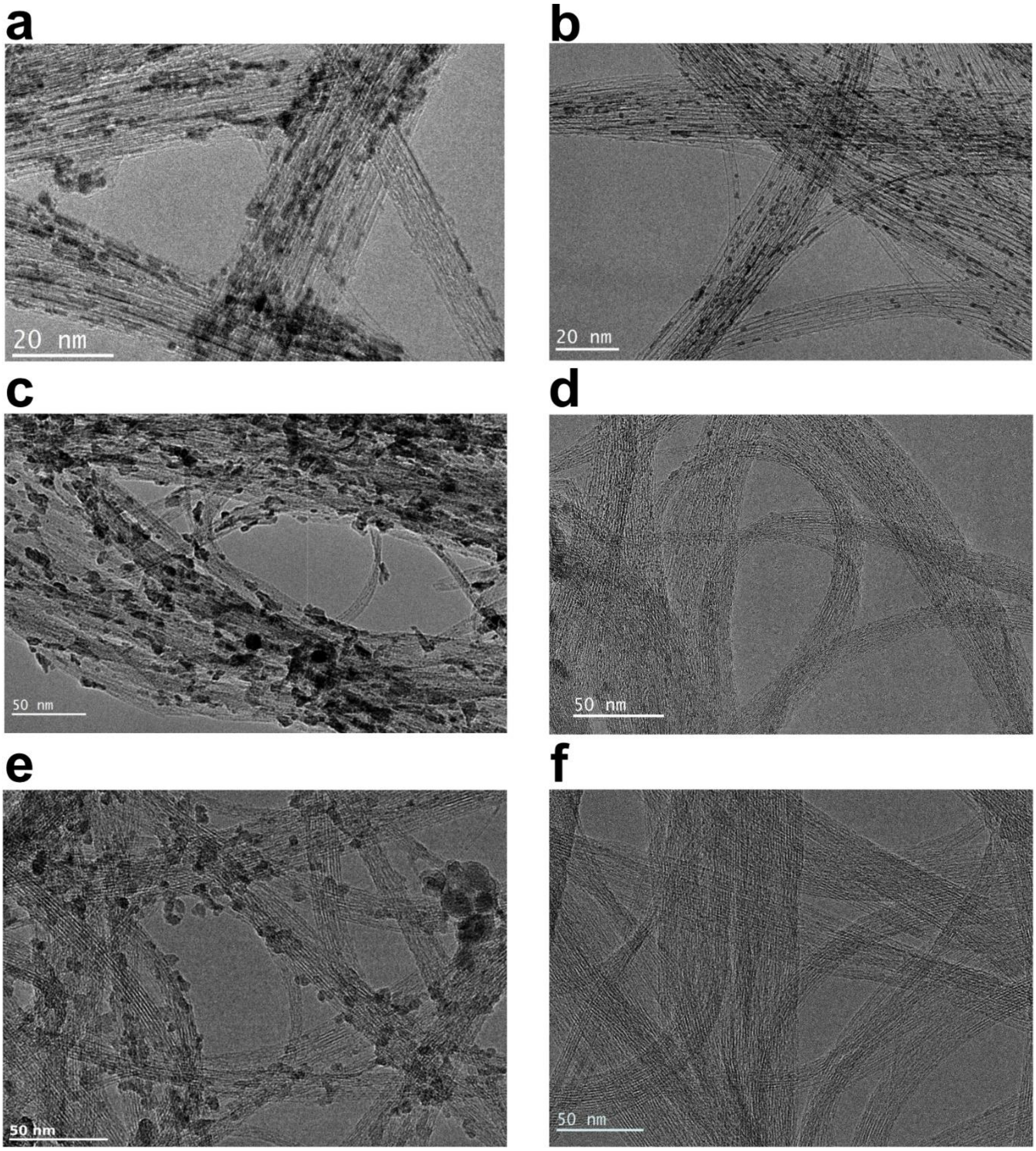

Figure S4 | TEM images of metal oxide and SWNT catalysts before and after washing. a) IrO_2/SWNT, b) IrO_2@SWNT, c) RuO_2/SWNT, d) RuO_2@SWNT, e) Co_3O_4/SWNT and f) Co_3O_4@SWNT. The surface nanoparticles present in images a, c and e (areas of darker contrast) are removed after washing, as seen in images b, d and f. All images were taken with a JOEL 2100F TEM field emission gun microscope operating at an accelerating voltage of 200 kV.

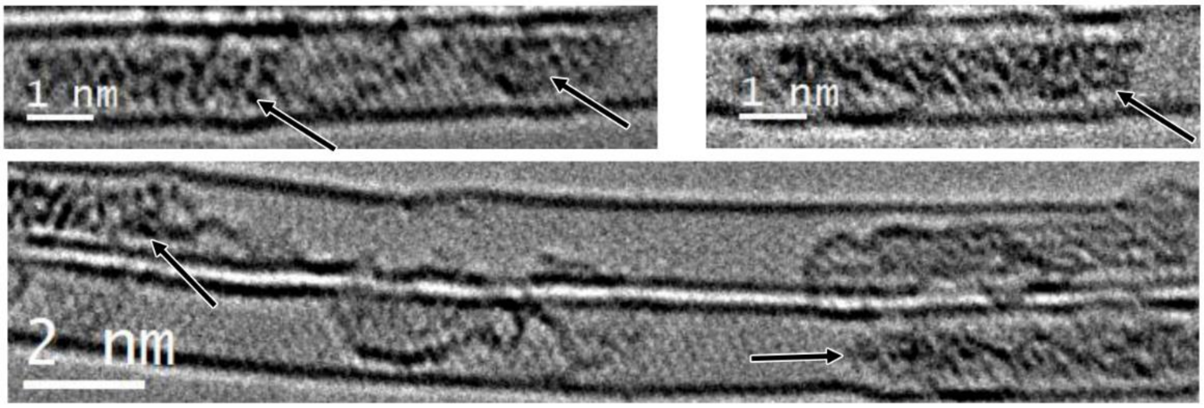

Figure S5 | AC-HRTEM images of Co_3O_4@SWNT. Arrows indicate encapsulated Co_3O_4 nanoparticles. AC-TEM images were acquired using the dedicated low voltage spherical (C_S) and chromatic (C_C) aberration corrected SALVE TEM microscope operated at 60 kV.

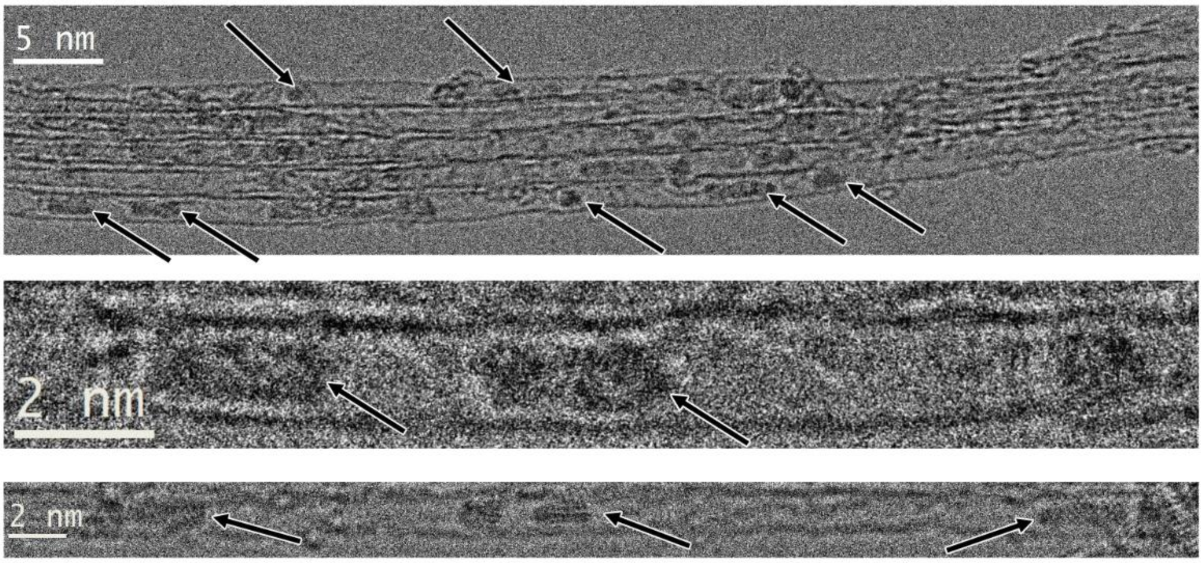

Figure S6 | HRTEM images of RuO_2@SWNT. Arrows indicate encapsulated RuO_2 nanoparticles. HR-TEM images were acquired using a JOEL 2100F TEM field emission gun microscope operated at 200 kV.

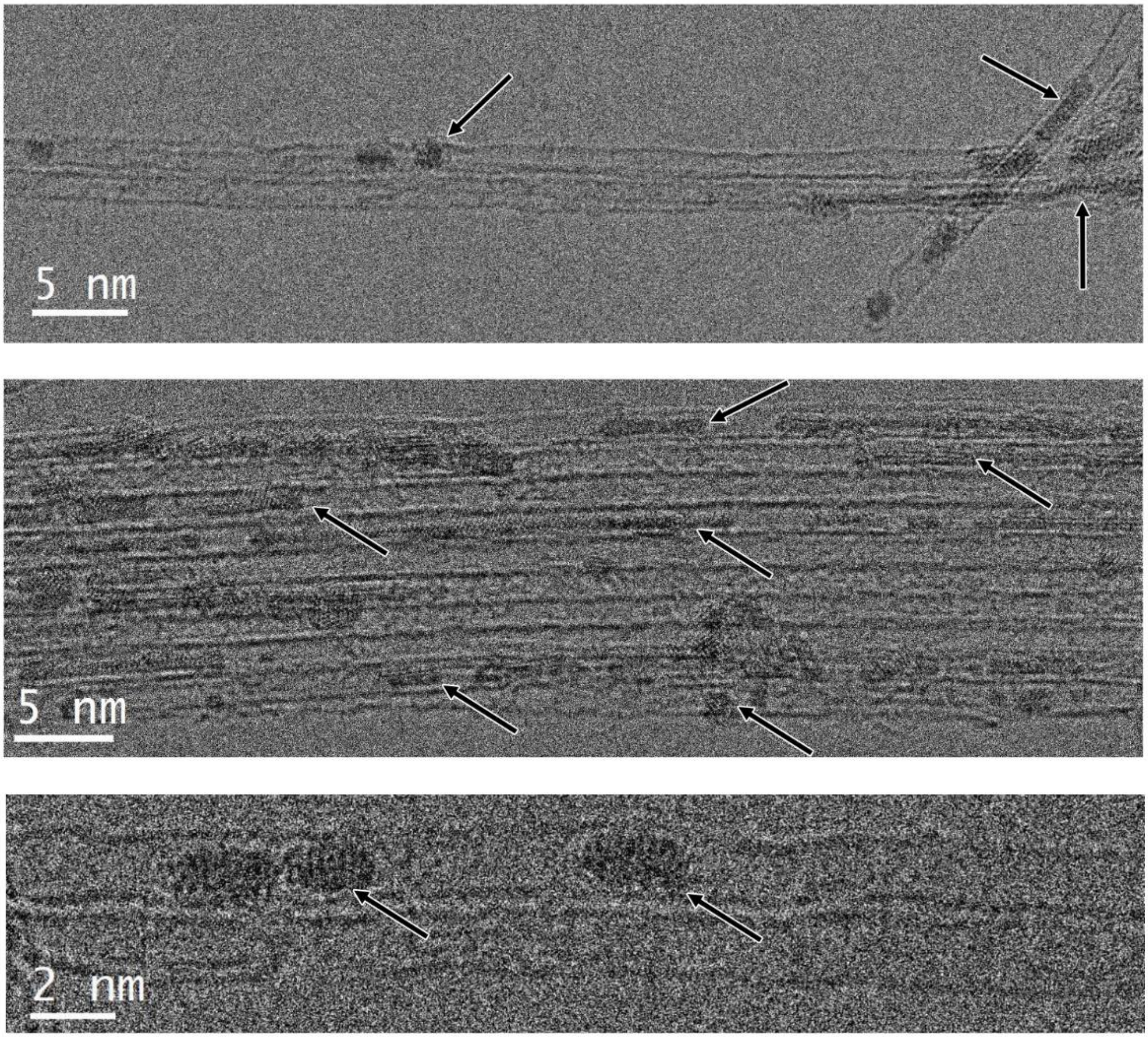

Figure S7 | HRTEM images of IrO_2@SWNT. Arrows indicate encapsulated IrO_2 nanoparticles. HR-TEM images were acquired using a JOEL 2100F TEM field emission gun microscope operated at 200 kV.

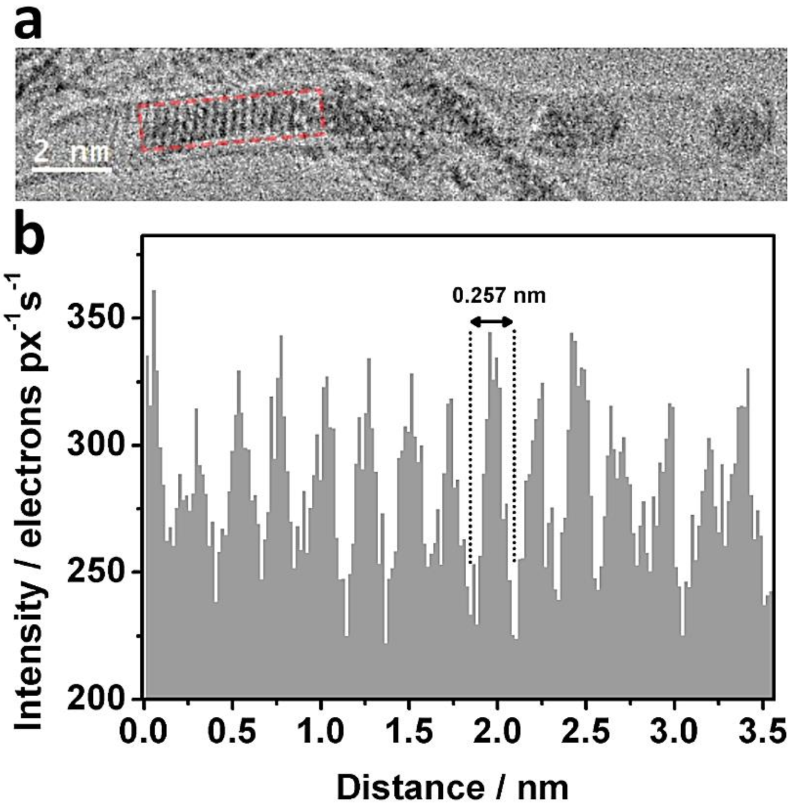

Figure S8 | d-spacing of encapsulated IrO_2@SWNT. a) HRTEM image of IrO_2@SWNT showing encapsulated IrO_2 (the same area is shown on the right-hand side of the top panel in Fig. S7). b) corresponding line profile of the contrast of the TEM image, taken from the highlighted area moving from left to right as shown in the image. Areas of low intensity indicate areas of high contrast, due to scattering of the electrons by the sample. A regular d-spacing of 0.257 ± 0.018 nm is apparent for the encapsulated IrO_2.

Supplementary note 1

For Co_3O_4@SWNT and RuO_2@SWNT, the crystal structure of the encapsulated species could not be determined from the TEM images under these imaging conditions due to electron beam-induced dynamics, a common phenomenon when analyzing metal oxides by TEM.[12,13] In the case of IrO_2@SWNT. However, the encapsulated IrO_2 was stable enough under the electron beam for d-spacing of 0.257 ± 0.018 nm to be seen (Fig. S8). Using Bragg's law, a d-spacing value of 0.257 nm gives a 2θ value of 34.8 degrees, which matches well with the (101) plane of rutile-type IrO_2.[14,15]

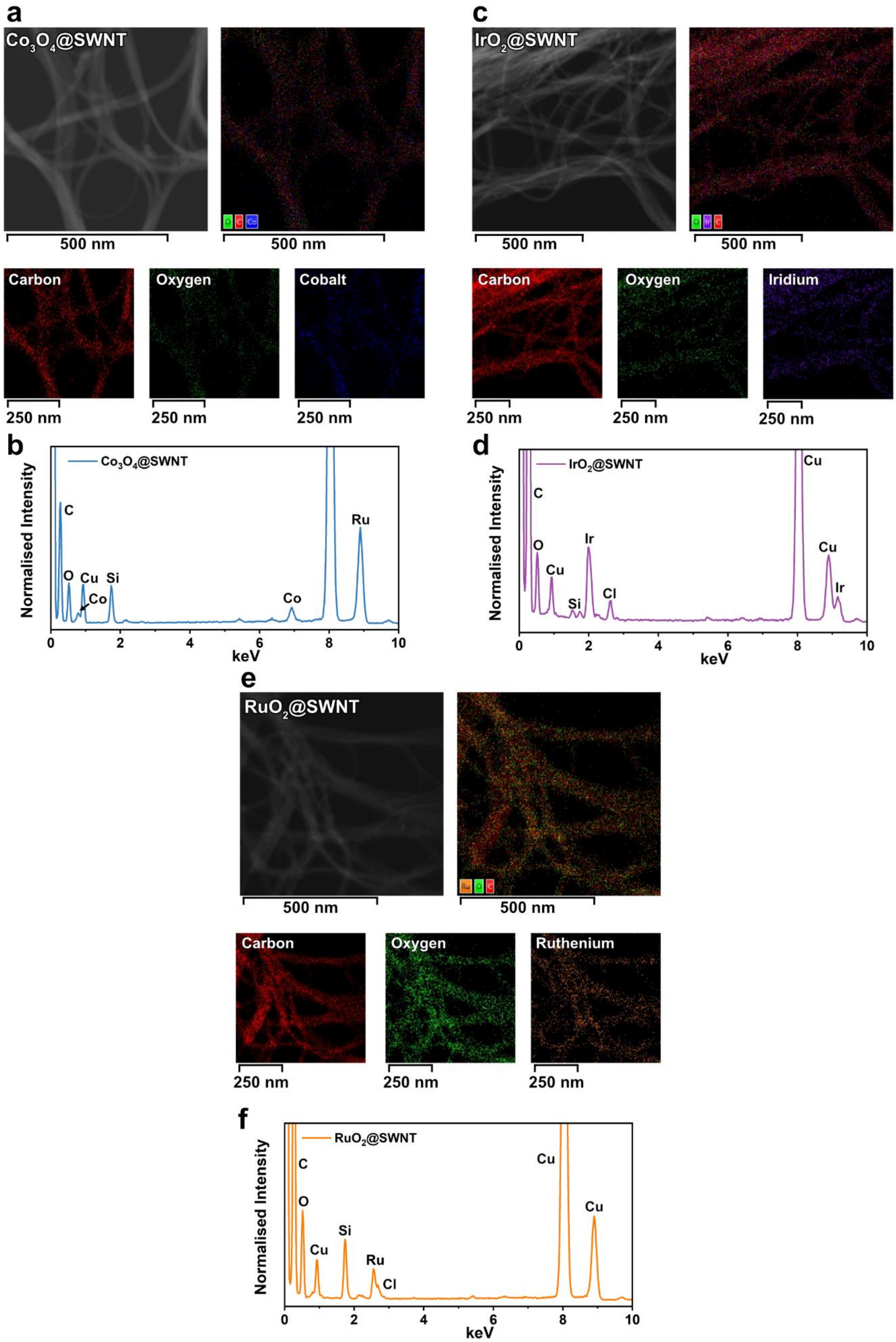

Figure S9 | Scanning transmission electron microscopy (STEM) energy dispersive X-ray (EDX) maps and corresponding spectra of Co_3O_4@SWNT, IrO_2@SWNT and RuO_2@SWNT. Small peaks at 2.1, 5.4 and 6.4 eV are due to gold, chromium and iron arising from the sample holder.

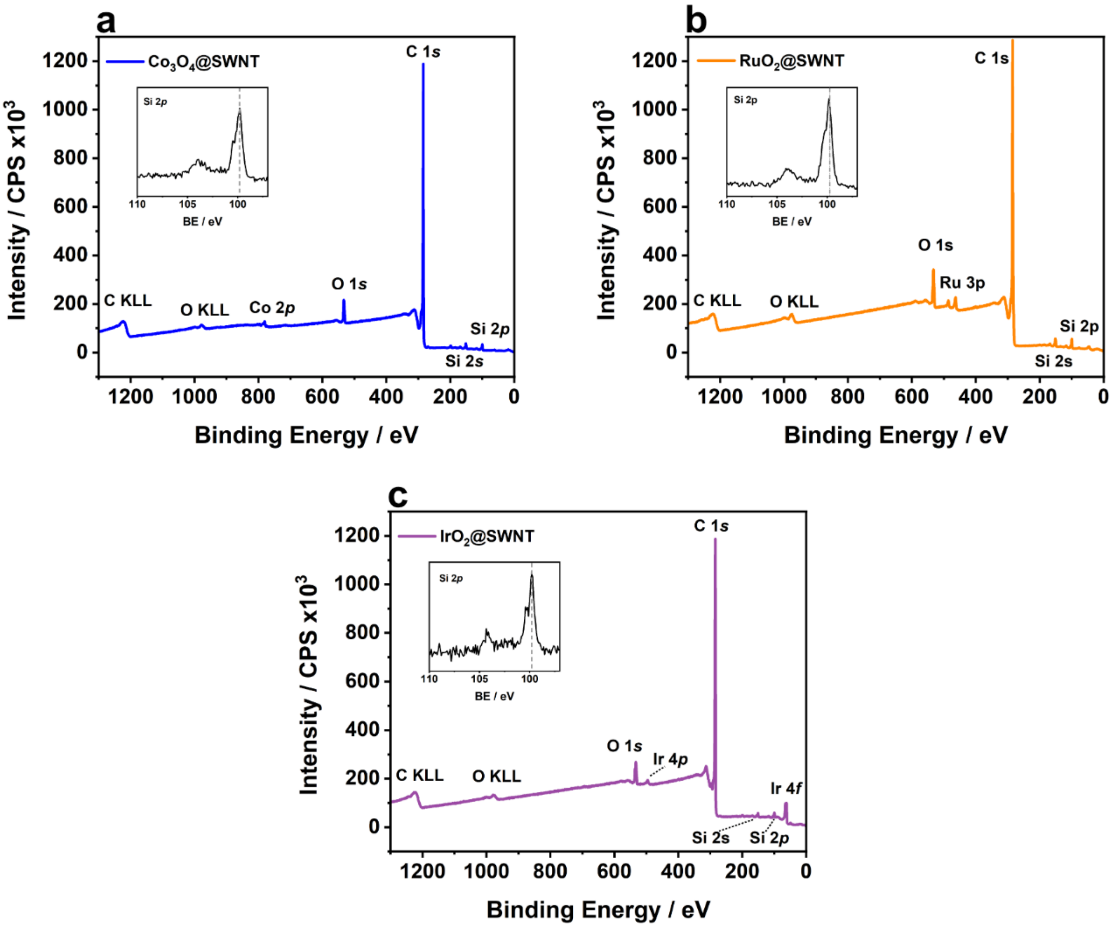

Figure S10 | XP spectra of a) Co_3O_4@SWNT, b) RuO_2@SWNT and c) IrO_2@SWNT. Inset is the high-resolution Si 2p region taken from each sample, originating from the Si wafer support, which is used here as an internal reference for charge correction.

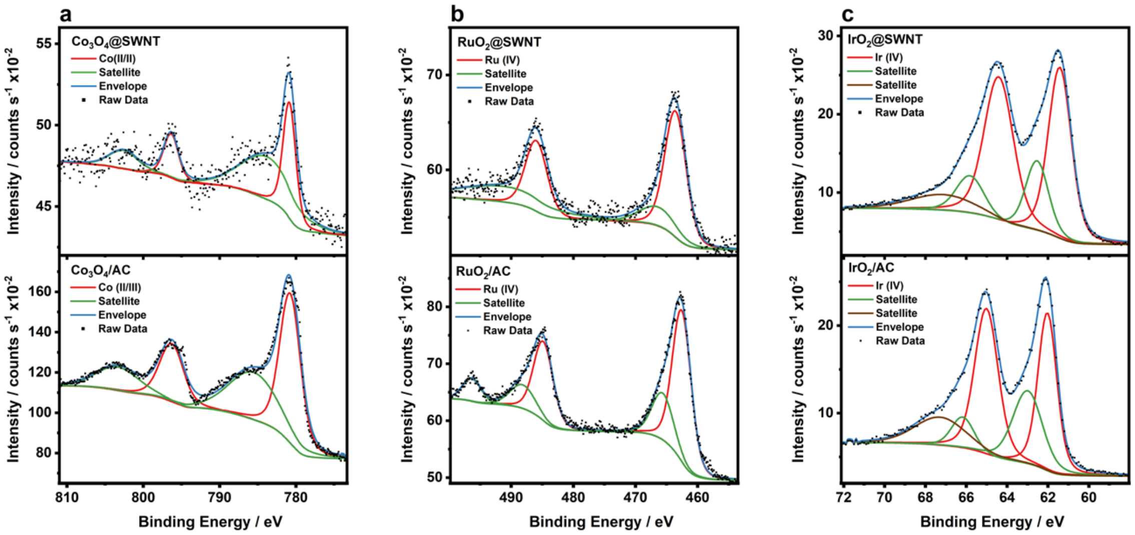

Figure S11 | XP spectra of a) Co 2p region of Co_3O_4@SWNT (top) and Co_3O_4/AC (bottom), b) Ru 3p region of RuO_2@SWNT (top) and RuO_2/AC (bottom) and c) Ir 4f region of IrO_2@SWNT (top) and IrO_2/AC (bottom).

Supplementary note 2

X-ray photoelectron spectroscopy (XPS) analysis of the MO_x@SWNT materials (Fig. S11) was performed to elucidate the state of confined MO_x. In Co_3O_4@SWNT, Co $2p_{1/2}$ and $2p_{3/2}$ peaks are seen at 796.4 eV and 781.0 eV, respectively, matching with literature values for Co_3O_4.[16,17] We note that the precise assignment of cobalt oxide species can be difficult with XPS due to overlapping peaks in the Co 2p peak, and it is possible this cobalt oxide species could be CoO_2 or CoO.[18] Two distinct peaks are seen within the $2p_{3/2}$ region at 780.1 eV and 781.7 eV, corresponding to Co^{3+} and Co^{2+}, respectively. The same is seen in the $2p_{1/2}$ region with peaks at 795.5 eV and 797.1.0 eV, corresponding to Co^{3+} and Co^{2+}, respectively.[19,20] Ru $3p_{3/2}$ and $3p_{1/2}$ values of 463.3 and 485.8 eV were seen, once again matching well to literature values for RuO_2. IrO_2@SWNT had Ir $4f_{7/2}$ and Ir $4f_{5/2}$ values of 61.4 and 64.4 eV respectively, matching well to literature values for IrO_2, corroborating the elucidation of IrO_2 by TEM analysis (supplementary note 1).[21] Direct comparison of the metal peaks between MO_x@SWNT and MO_x/AC materials is difficult with both charge transfer from the carbon and changes in metal oxide morphology affecting the shape and position of the peaks. Metal peaks in the MO_x/AC materials are observed at similar positions as MO_x@SWNT (Figure S11), indicating the presence of Co_3O_4, RuO_2 and IrO_2. In the case of IrO_2/AC, a shift to higher binding energies as compared to IrO_2@SWNT is observed, which may be due to charge transfer from the SWNT to the encapsulated IrO_2.

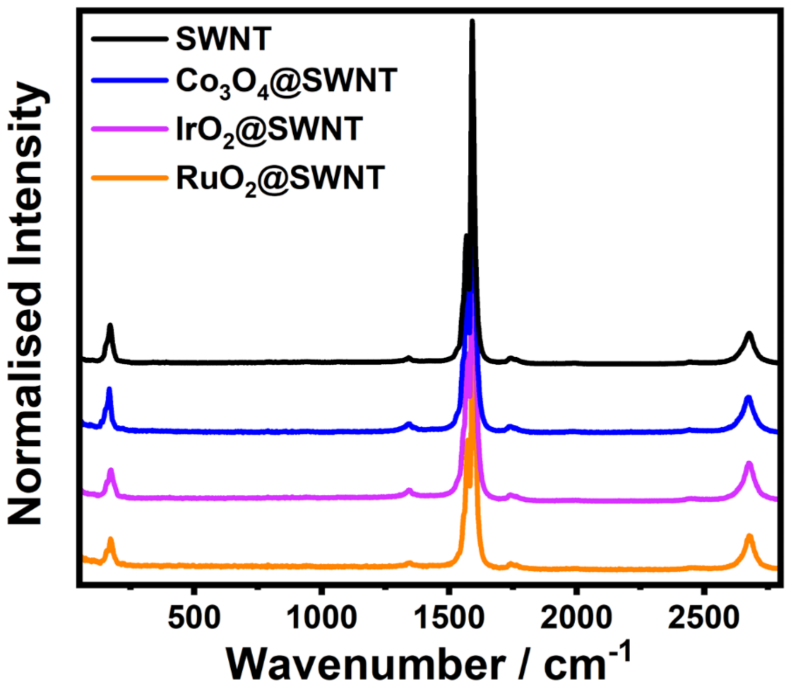

Figure S12 | 532 nm resonance Raman spectra of SWNTs, Co_3O_4@SWNT, IrO_2@SWNT and RuO_2@SWNT, showing the RBM, D and G bands of semiconducting SWNT at ~170 cm^{-1}, 1330 cm^{-1} and 1600 cm^{-1}, respectively.

Table S2 | Electrochemical performance of the MO_x@SWNT catalysts. The overpotential required to reach 10 mA cm^{-2}, η_{10}, and Tafel slopes are taken from LSVs performed in N_2 saturated 0.1 M potassium hydroxide using a scan rate of 5 mV/s for each catalyst (Fig. 2a) before and after electrochemical cycling between 0.9 and 1.7 V using a scan rate of 100 mV/s for 2000 cycles in N_2 saturated 0.1 M potassium hydroxide.

Catalyst	Change in η_{10} after 2000 cycles / mV	Tafel slope / mV dec^{-1}	Tafel slope after 2000 cycles / mV dec^{-1}
**Co_3O_4@SWNT**	14	100	83
**RuO_2@SWNT**	3	192	67
**IrO_2@SWNT**	-25	89	77
**SWNT**	N/A	108	159

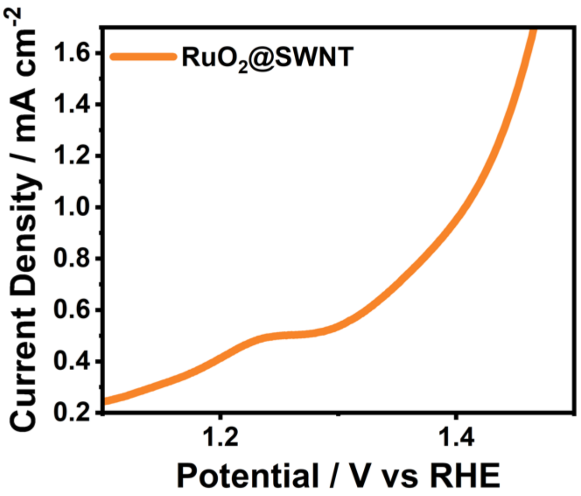

Figure S13 | LSV of RuO_2@SWNT in O_2 saturated 0.1M potassium hydroxide, showing RuO_2 oxidation at 1.24 V vs RHE.

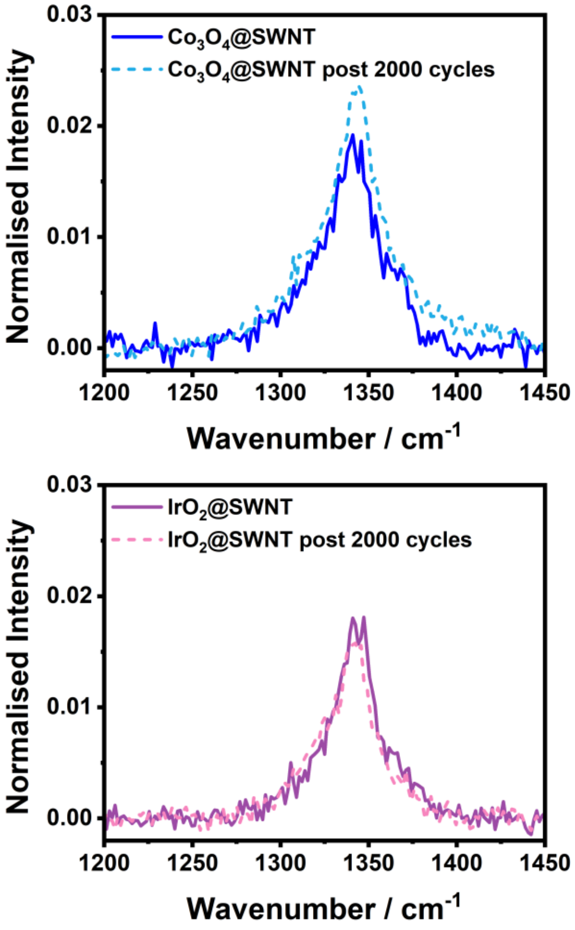

Figure S14 | (a-d) Raman spectra of Co_3O_4@SWNT, RuO_2@SWNT, IrO_2@SWNT and SWNT respectively, before and after cycling 2000 times in 0.1 M KOH. Spectra highlight the D band, with intensities normalised to the G band.

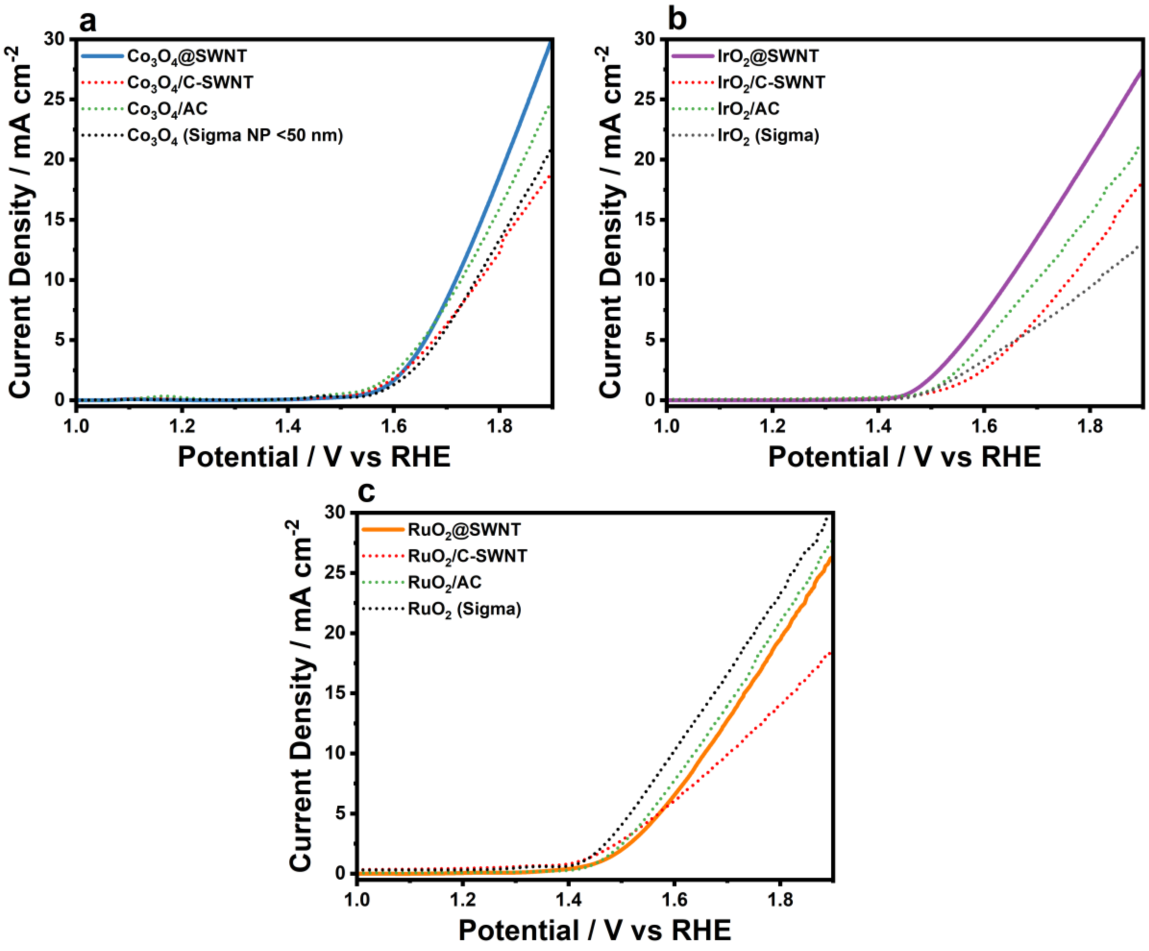

Figure S15 | a-c) LSVs of Co_3O_4@SWNT, IrO_2@SWNT and RuO_2@SWNT respectively, compared to relevant MO_x/C-SWNT, MO_x/AC and commercially available MO_x in O_2 saturated 0.1 M potassium hydroxide.

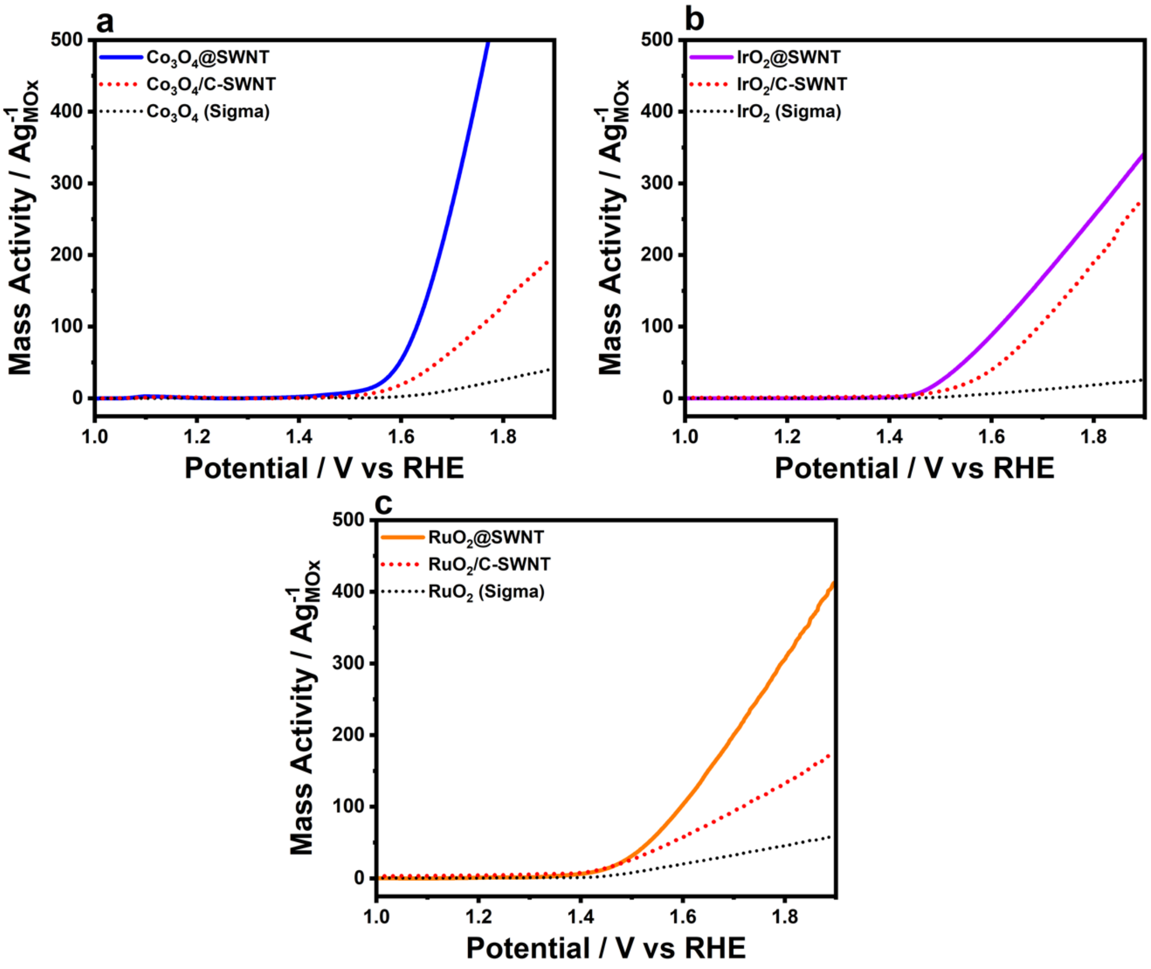

Figure S16 | a-c) LSVs presented as mass activities of Co_3O_4@SWNT, IrO_2@SWNT and RuO_2@SWNT respectively, compared to relevant MO_x/C-SWNT, and commercially available MO_x in O_2 saturated 0.1 M potassium hydroxide. Mass activity is calculated based on weight of MO_x drop-cast on the electrode using relevant TGA data (Fig. S1 and S17).

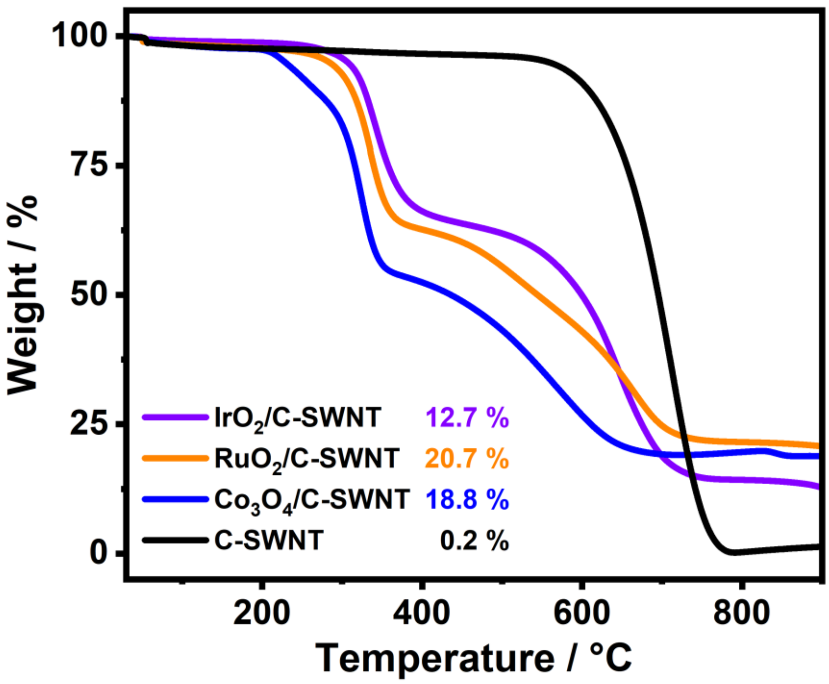

Figure S17 | Thermogravimetric analysis (TGA) traces of all MO_x/C-SWNT materials. The final weight loadings are given as a weight %.

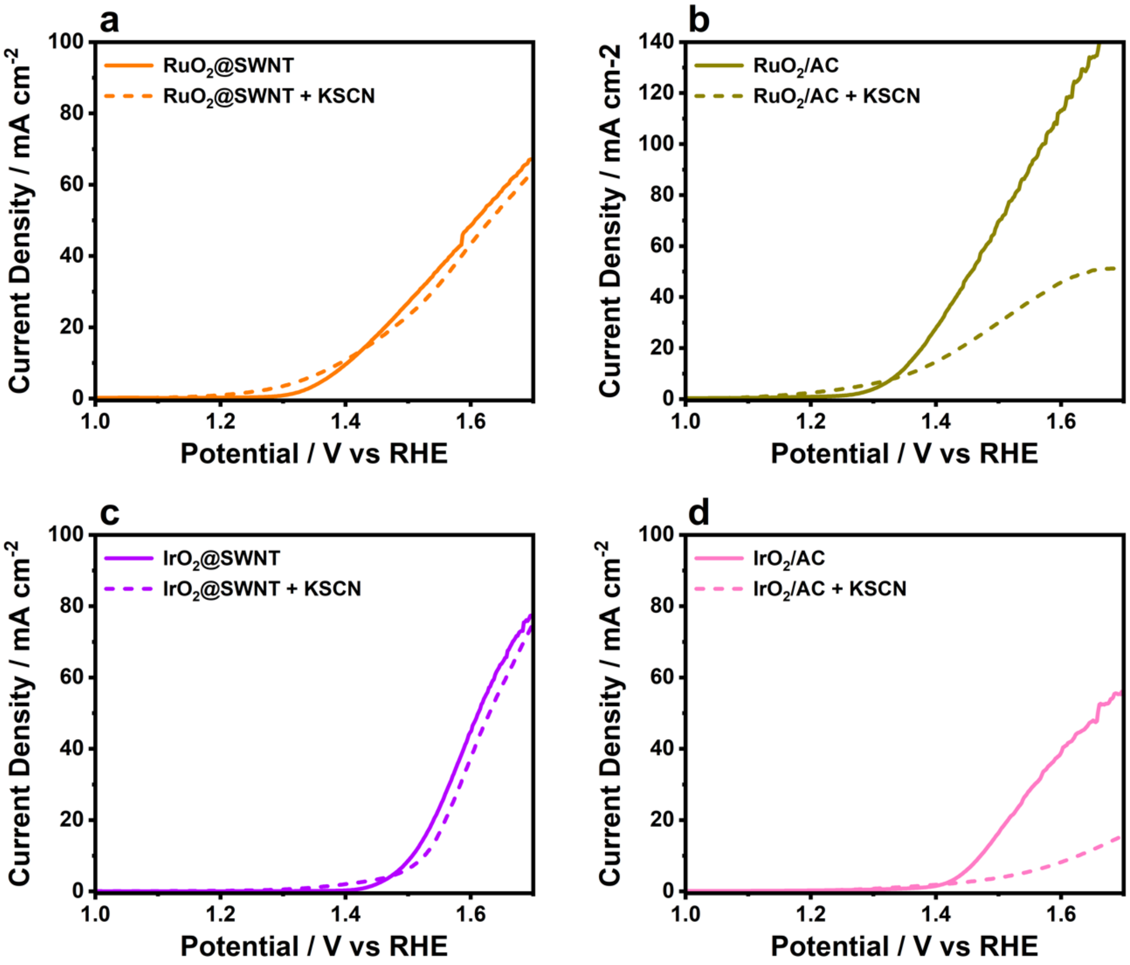

Figure S18 | KSCN poisoning experiments of RuO_2@SWNT, RuO_2/AC, IrO_2@SWNT and IrO_2/AC (a-d) respectively. LSVs were performed in 1.0 M KOH using a scan rate of 5 mV/s, before addition of 0.1 M KSCN. The modified working electrode was allowed to rotate in 1.0 M KOH and 0.1 M KSCN for 30 minutes before another LSV was performed.

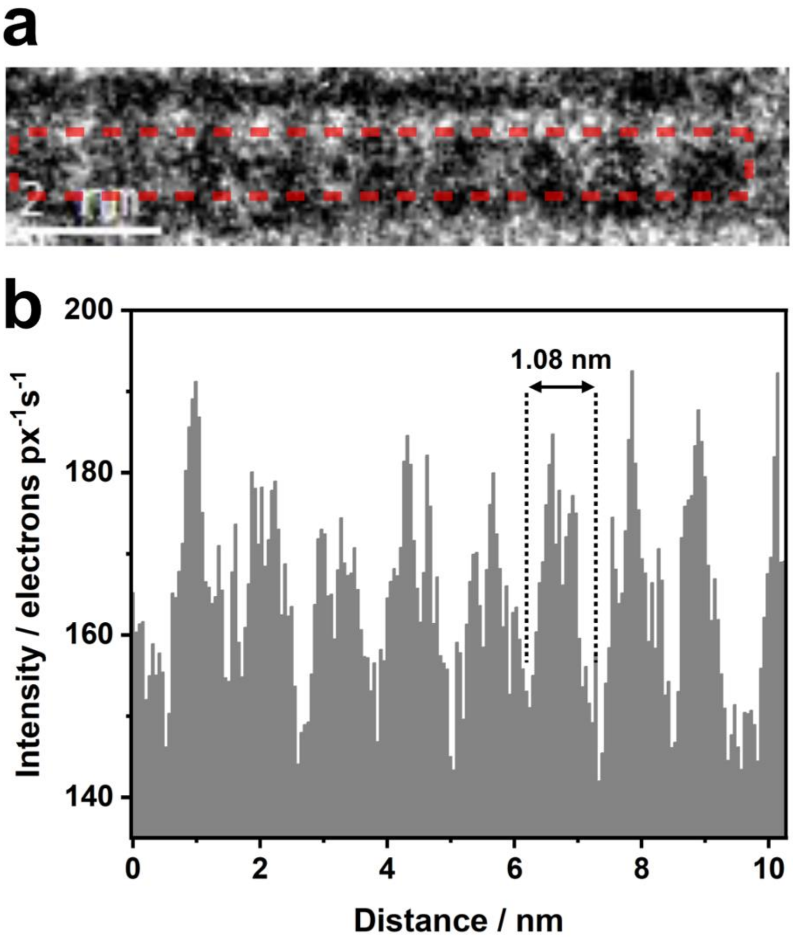

Figure S19 | TEM analysis of (C_{60})-Co_3O_4@SWNT. a) HRTEM image showing encapsulated C_{60} at tube ends and b) Line profile (highlighted in image a) showing inter-C_{60} distances of 1.08±0.05 nm, which match well to literature values for inter-C_{60} distances in C_{60}@SWNT.

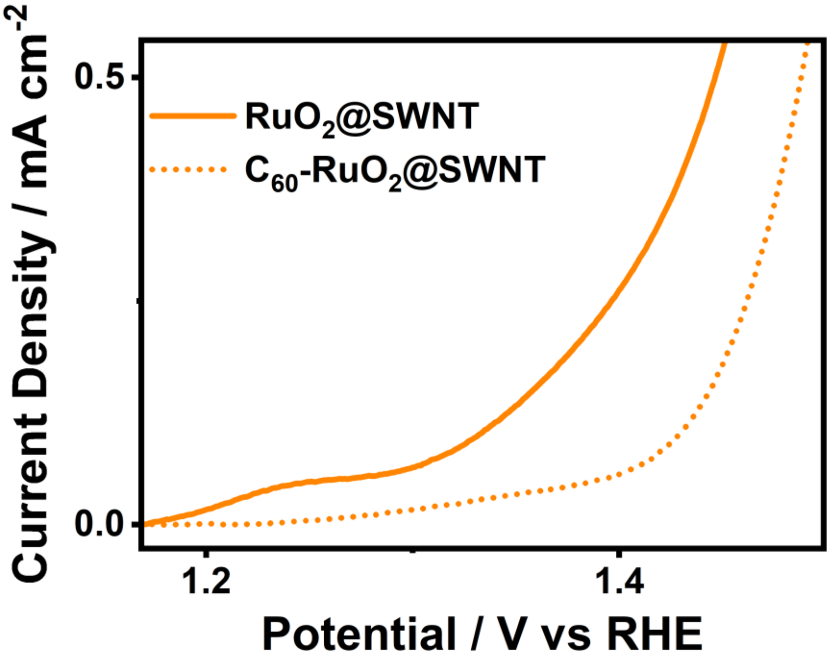

Figure S20 | LSV of RuO_2@SWNT and C_{60}-RuO_2@SWNT in N_2 saturated 0.1M potassium hydroxide, showing the RuO_2 oxidation at 1.24 V vs RHE and the loss of oxidation process upon capping.

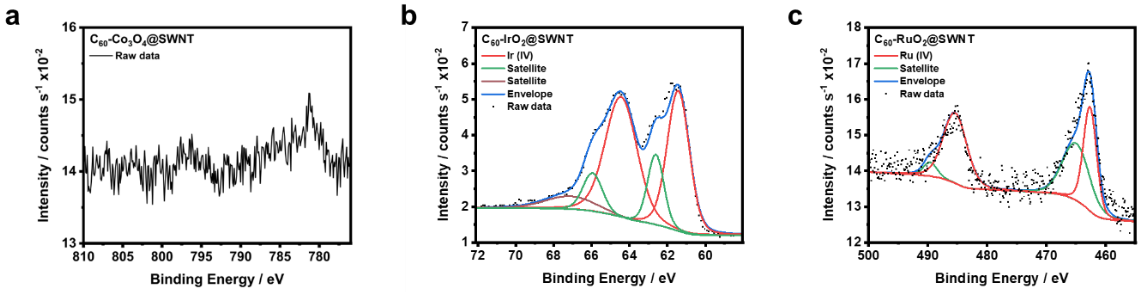

Figure S21 | XPS spectra of a) Co 2p region of Co_3O_4@SWNT b) 4f region of IrO_2@SWNT and c) 3p region of RuO_2@SWNT plugged with C_{60} after polarisation of 0 to 1.6 V vs RHE in N_2 saturated 0.1 M KOH at 5 mV s^{-1}.

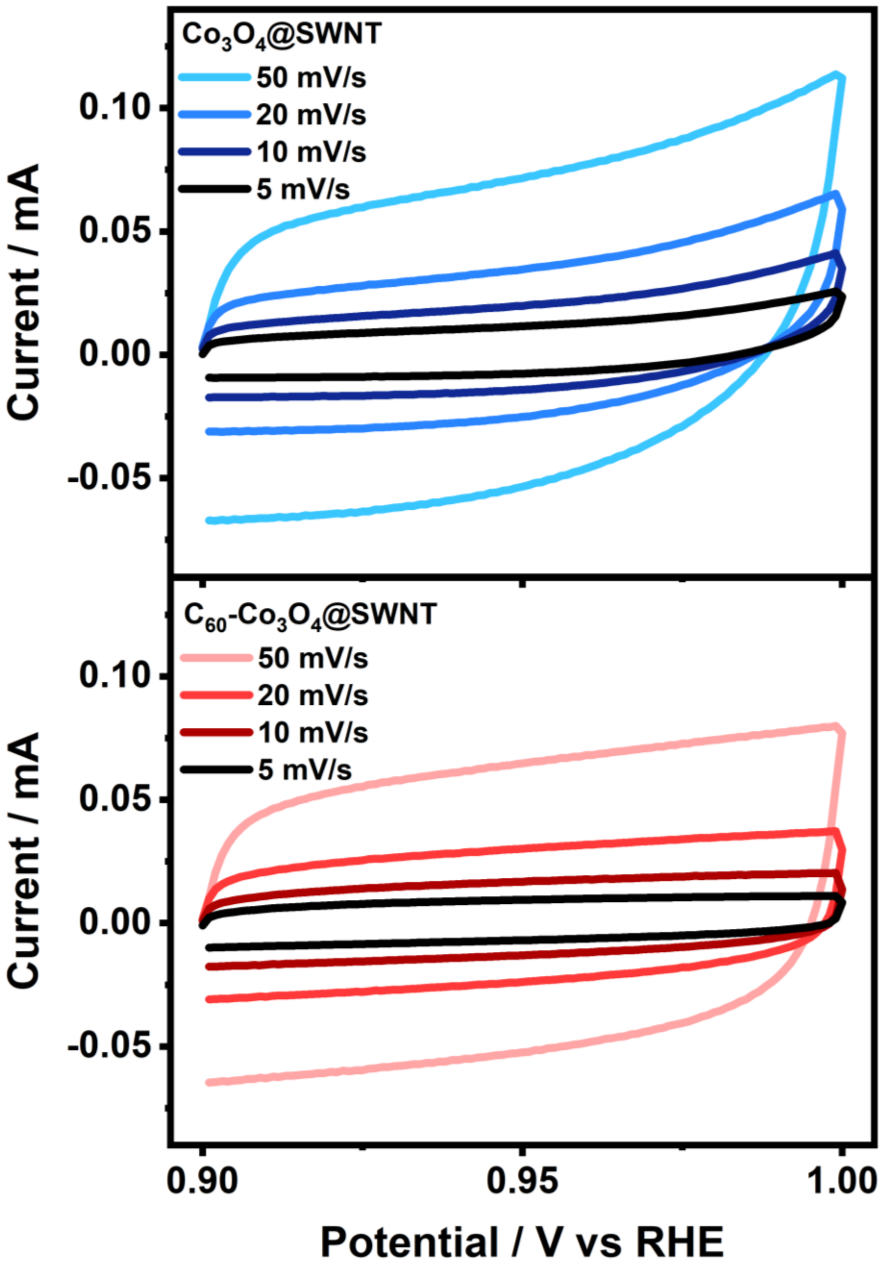

Figure S22 | Cyclic voltammograms of Co_3O_4@SWNT and C_{60}-Co_3O_4@SWNT in a non-faradaic region at different scan rates, recorded in N_2 saturated 0.1 M potassium hydroxide. Current values were taken at 0.95 V vs RHE and the capacitance was calculated from the slope of a plot of scan rate against current to give a value of 1.16 mF and 1.10 mF for Co_3O_4@SWNT and C_{60}-Co_3O_4@SWNT, respectively.

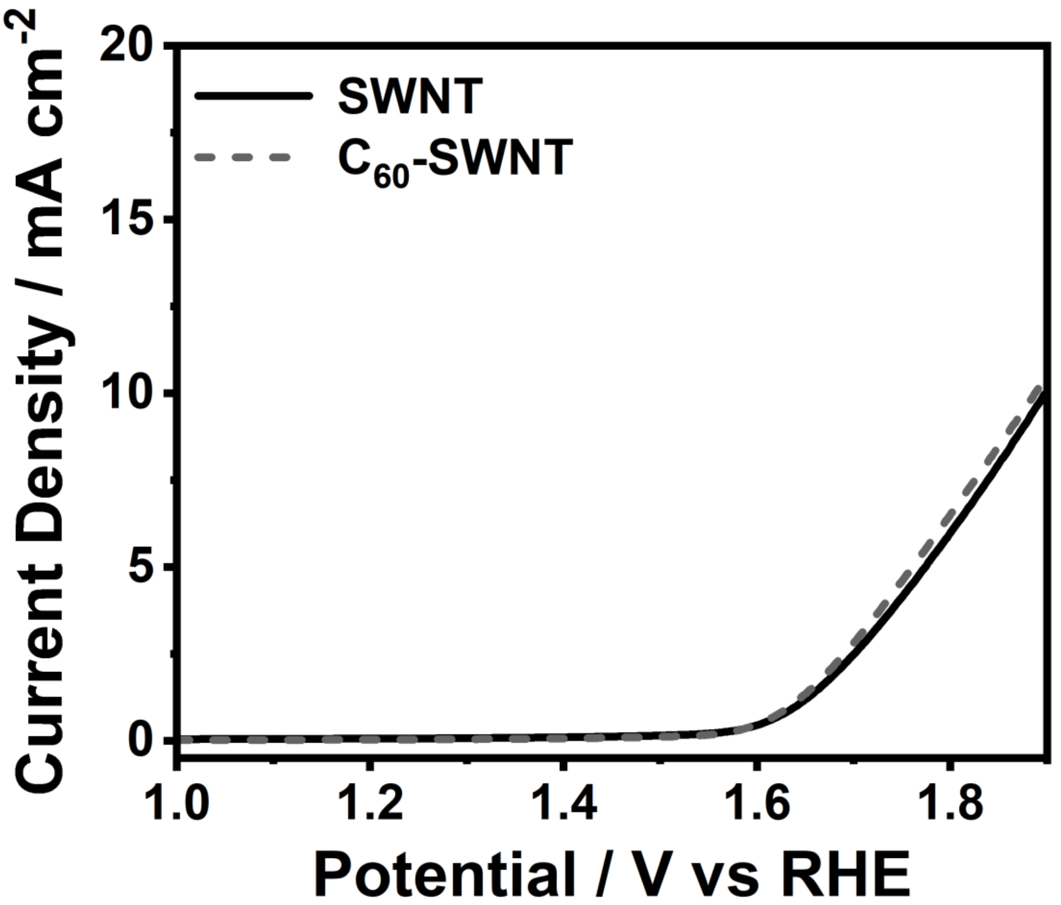

Figure S23 | RDE-LSVs of SWNT and C_{60}-SWNT, recorded in N_2 saturated 0.1 M potassium hydroxide at 5 mV s^{-1} and 1600 rpm.

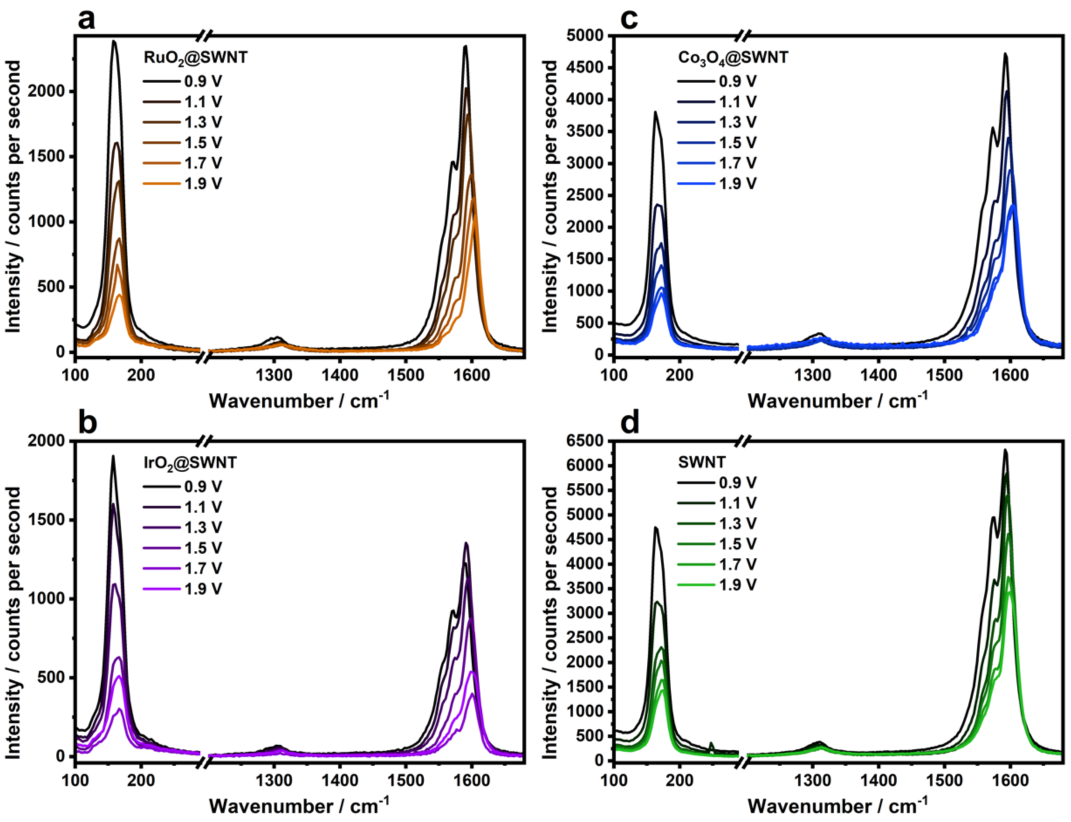

Figure S24 | 785 nm Raman spectra of MOx@SWNT materials, taken at potential holds from 0.9 to 1.9 V vs RHE in N_2 saturated 0.1 M potassium hydroxide. Each spectrum shown represents the average from 9 spectra collected from a 50x50 um regular array across the modified electrode surface.

Table S3 | Changes in I_D:I_G and G band position for MO_x@SWNT between 0.9 V and 1.9 V vs RHE in 0.1 M potassium hydroxide, taken from in-situ electrochemical 785 nm Raman spectroscopy (Fig. S24).

	Increase in I_D:I_G / %	Blueshift in position of G band / cm^{-1}
**Co_3O_4@SWNT**	28	12
**RuO_2@SWNT**	2	14
**IrO_2@SWNT**	28	9
**SWNT**	22	7

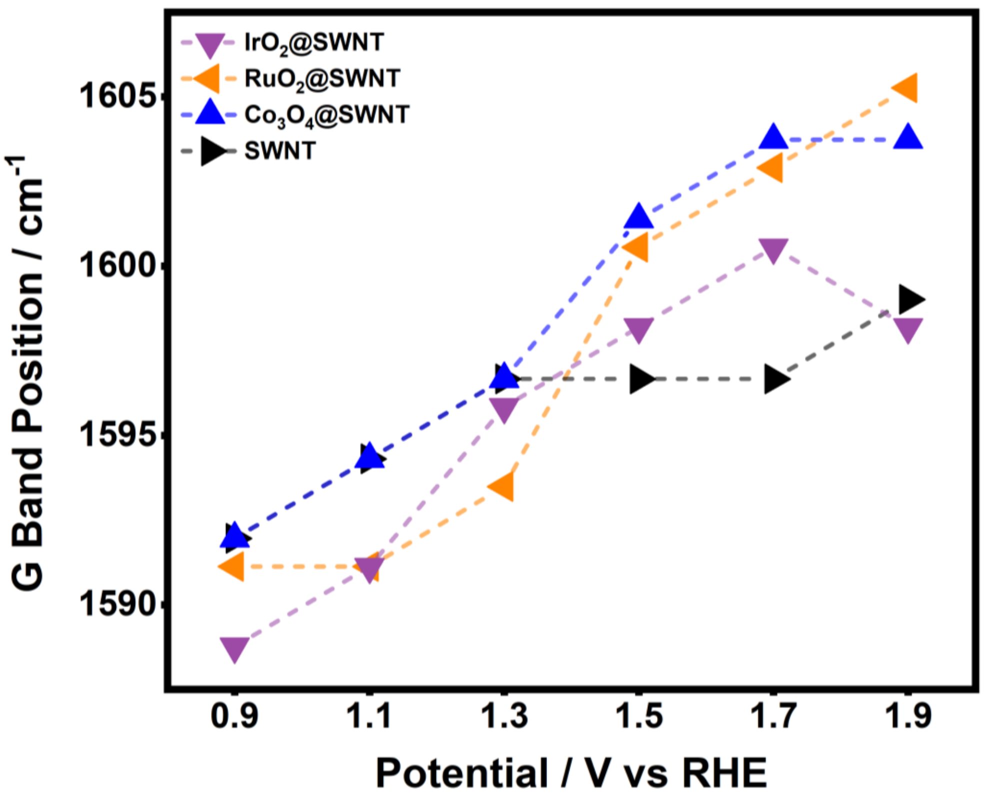

Figure S25 | G band position of each catalyst as a positive potential is applied, taken from the 785 nm Raman spectra shown in Fig. S24.

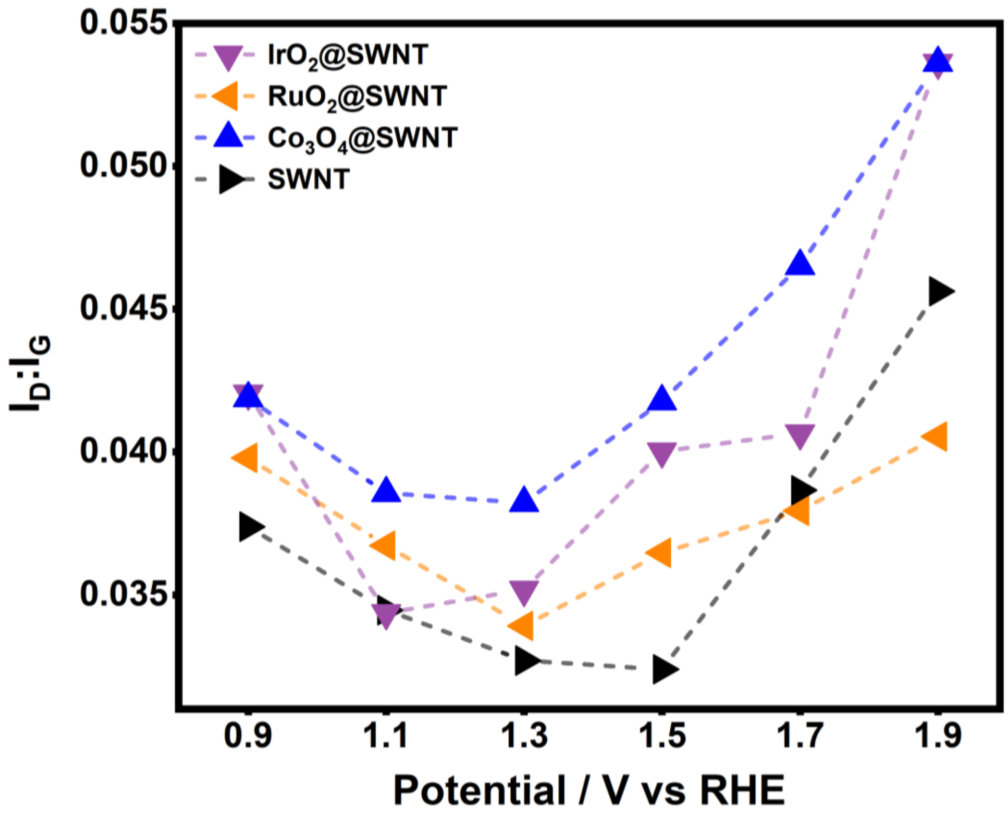

Figure S26 | $I_D:I_G$ of Co_3O_4@SWNT, RuO_2@SWNT, IrO_2@SWNT and SWNTs as a positive potential is applied, taken from the 785 nm Raman spectra shown in Fig. S24.

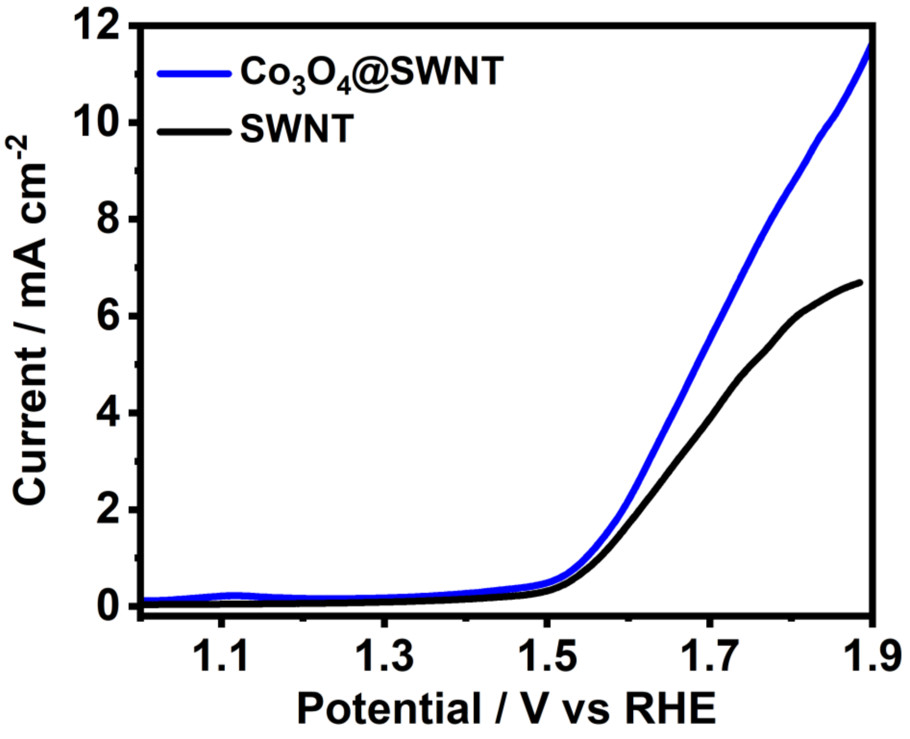

Figure S27 | LSV of Co_3O_4@SWNT and SWNTs, taken before in-situ electrochemical Raman spectroscopy.

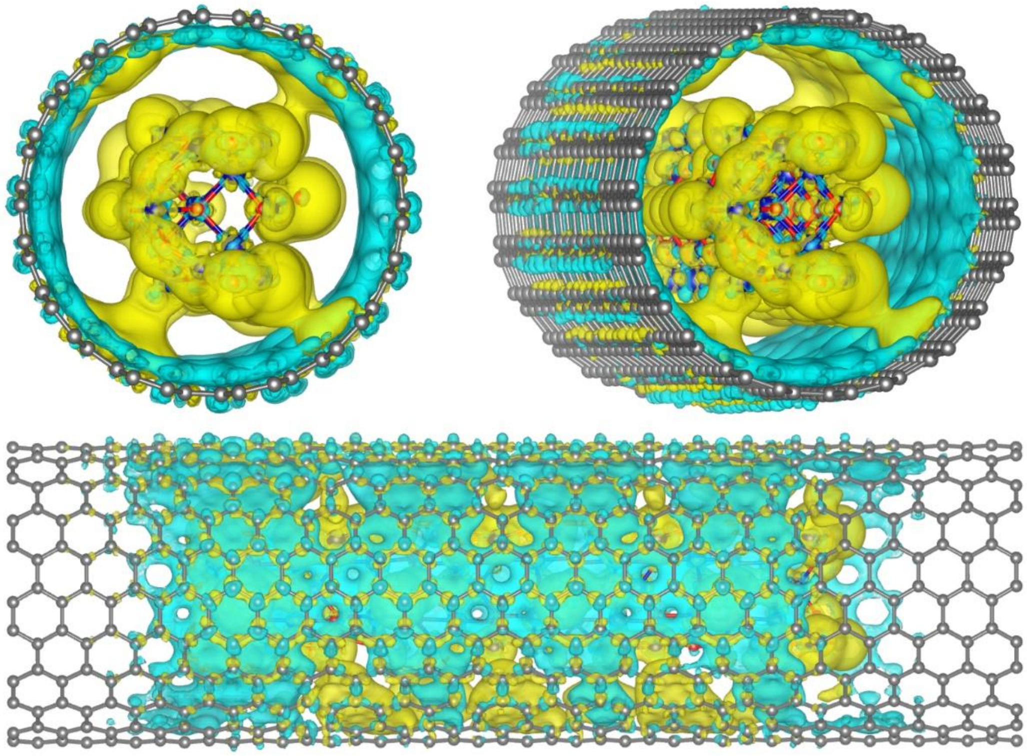

Figure S28 | Electronic density difference after the encapsulation of Co_3O_4@SWNT (11,11). Blue (yellow) colour represents the depletion (augmentation) of electronic density.

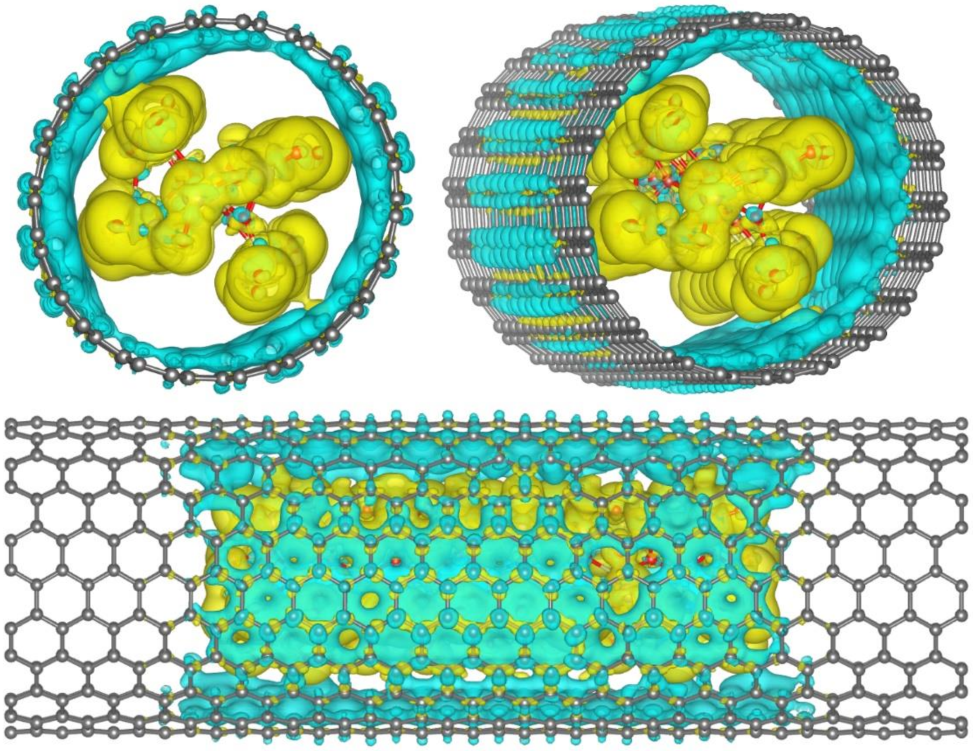

Figure S29 | Electronic density difference after the encapsulation of IrO_2@SWNT (11,11). Blue (yellow) colour represents the depletion (augmentation) of electronic density.

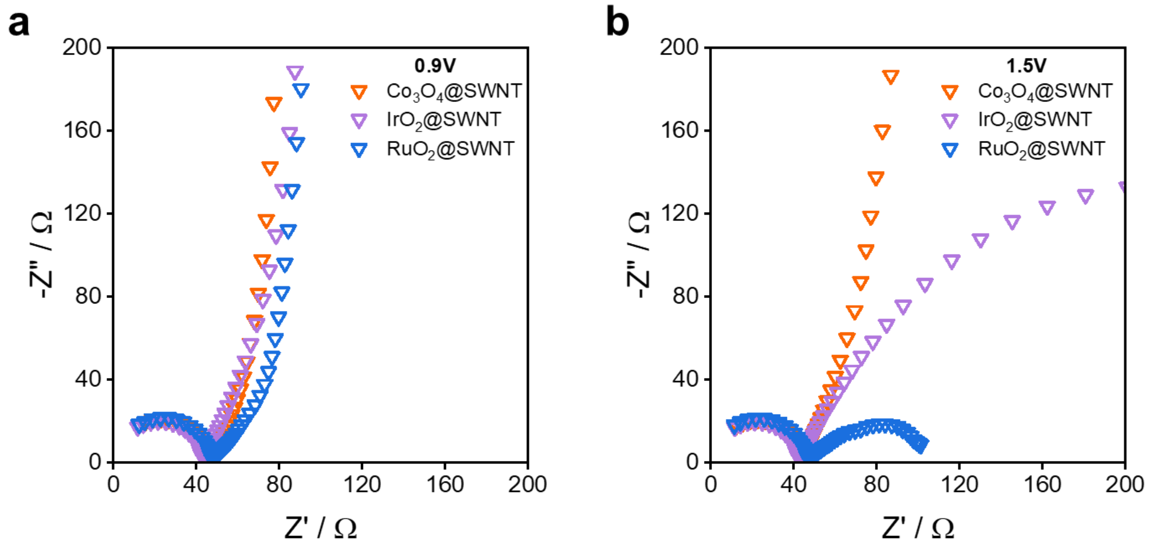

Figure S30 | Nyquist plots of SWNT, Co_3O_4@SWNT, IrO_2@SWNT and RuO_2@SWNT, taken at **a)** 0.9 V and **b)** 1.5 V vs RHE in N_2 saturated 0.1 M KOH, using frequencies between 100 kHz and 0.1 Hz at 1600 rpm.

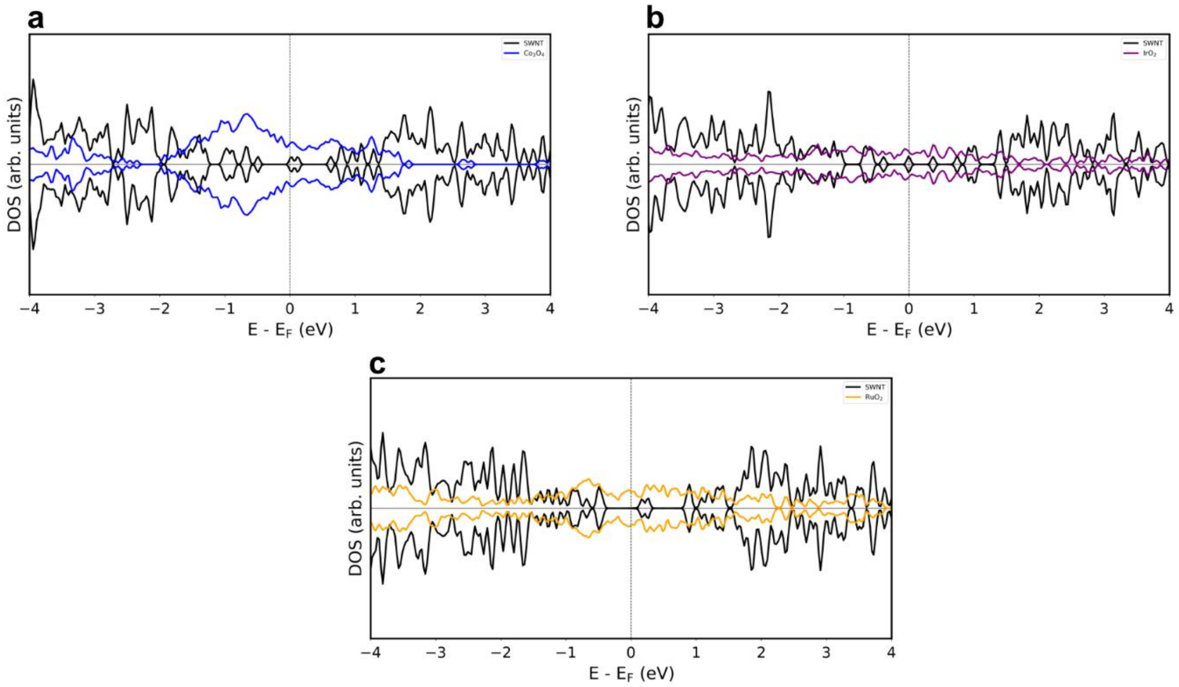

Figure S31 | Calculated density of states of Co_3O_4@SWNT, IrO_2@SWNT and RuO_2@SWNT.

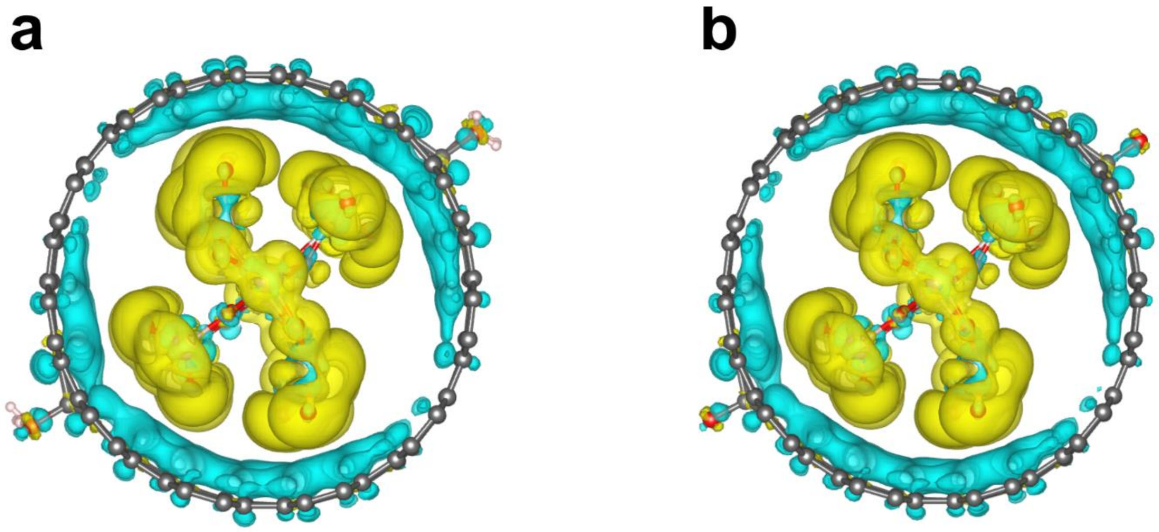

Figure S32 | Electronic density difference after the encapsulation of RuO_2 on SWNT (11,11) with attached -OH groups (a) and -O groups (b). Blue (yellow) colour represents the depletion (augmentation) of electronic density.

Table S4 | Mulliken atomic charges of modified nanotubes, showing charges on neighboring carbon atoms to attached oxygen groups.

	Mulliken atomic charges (a.u.)		
	RuO_2@CNT	-O	-OH
**C1**	0.013	0.033	0.003
**C2**	0.012	0.051	-0.006
**C3**	0.002	0.052	0.039
**C4**	0.003	0.044	0.036
**C5**	0.009	0.016	0.008
**C6**	0.007	0.025	0.003
**C7**	0.003	0.010	0.009
**C8**	0.003	0.013	0.014
**C9**	0.007	0.026	0.023
**C10**	0.004	0.017	0.013
**C11**	0.003	0.020	0.009
**C12**	0.002	0.017	0.010
**C13**	0.004	0.018	0.004

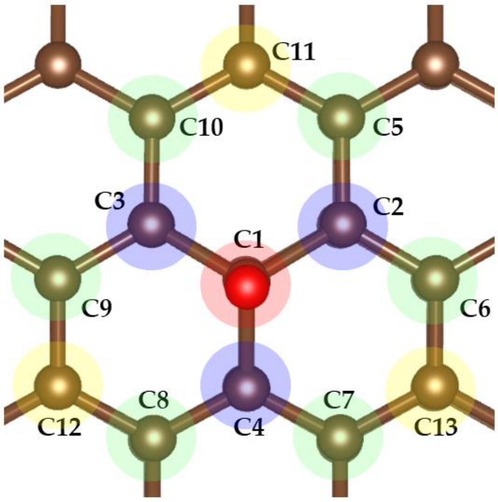

Supplementary note 3

To see how oxygen groups that would be introduced during a typical OER cycle could impact the electron density of neighbouring carbon atoms in these MO_x@SWNT systems, -O and -OH groups were attached to the carbon surface. RuO_2@SWNT system was chosen as a case of study as it was the material that showed the largest charge transfer in the pristine system. Mulliken atomic charges for neighbouring carbon atoms, are shown in Table S4. The highest changes of atomic charges of neighbouring carbon atoms are seen with attached -O groups, on the nearest neighbouring carbon atoms (blue shaded), while the effect on the second and third row of neighbouring C atoms is consistent but with less intensity. This increase in atomic charge could provide a lower activation barrier for a new OH^- binding process, thus lowering the onset

potential. This is consistent with Tafel analysis and electrochemical stability measurements, in which the MO_x@SWNT materials improve in OER catalytic activity, accompanied by a lowering in the Tafel slope after cycling.

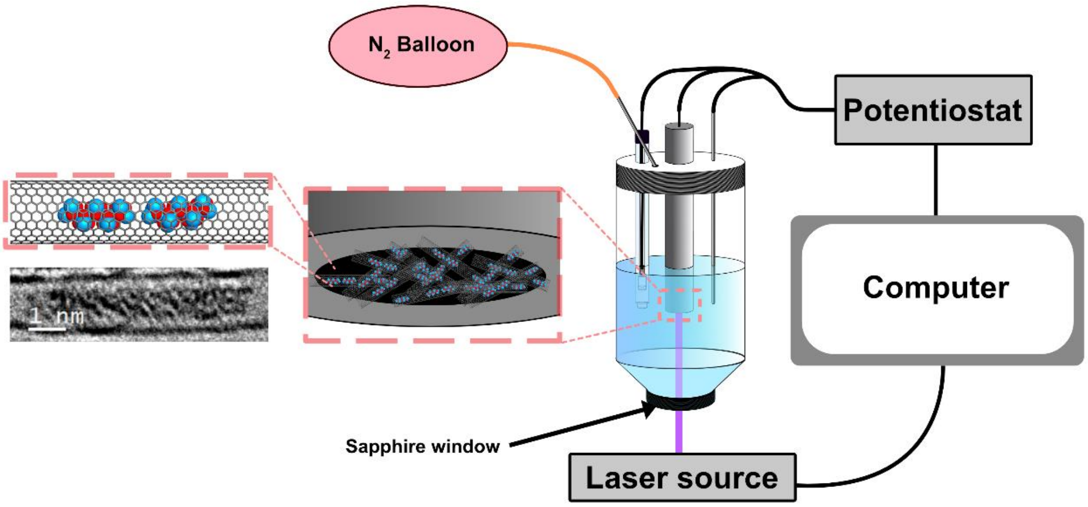

Figure S33 | Schematic of the cell used for Raman spectroelectrochemistry measurements. Further experimental details can be found the in the methods section.

Supplementary References

1 H. Z. Geng, X. B. Zhang, S. H. Mao, A. Kleinhammes, H. Shimoda, Y. Wu and O. Zhou, *Chemical Physics Letters*, 2004, **399**, 109–113.

2 S. Bandow, M. Takizawa, K. Hirahara, M. Yudasaka and S. Iijima, *Chemical Physics Letters*, 2001, **337**, 48–54.

3 D. S. Jensen, S. S. Kanyal, N. Madaan, M. A. Vail, A. E. Dadson, M. H. Engelhard and M. R. Linford, *Surface Science Spectra*, 2013, **20**, 36–42.

4 T. D. Kühne, M. Iannuzzi, M. Del Ben, V. V. Rybkin, P. Seewald, F. Stein, T. Laino, R. Z. Khaliullin, O. Schütt, F. Schiffmann, D. Golze, J. Wilhelm, S. Chulkov, M. H. Bani-Hashemian, V. Weber, U. Borštnik, M. Taillefumier, A. S. Jakobovits, A. Lazzaro, H. Pabst, T. Müller, R. Schade, M. Guidon, S. Andermatt, N. Holmberg, G. K. Schenter, A. Hehn, A. Bussy, F. Belleflamme, G. Tabacchi, A. Glöß, M. Lass, I. Bethune, C. J. Mundy, C. Plessl, M. Watkins, J. VandeVondele, M. Krack and J. Hutter, *The Journal of Chemical Physics*, 2020, **152**, 194103.

5 J. Hutter, M. Iannuzzi, F. Schiffmann and J. VandeVondele, *WIREs Computational Molecular Science*, 2014, **4**, 15–25.

6 J. P. Perdew, K. Burke and M. Ernzerhof, *Phys. Rev. Lett.*, 1996, **77**, 3865–3868.

7 C. Hartwigsen, S. Goedecker and J. Hutter, *Phys. Rev. B*, 1998, **58**, 3641–3662.

8 S. Goedecker, M. Teter and J. Hutter, *Phys. Rev. B*, 1996, **54**, 1703–1710.

9 J. VandeVondele and J. Hutter, *J Chem Phys*, 2007, **127**, 114105.

10 S. Grimme, J. Antony, S. Ehrlich and H. Krieg, *The Journal of Chemical Physics*, 2010, **132**, 154104.

11 F. L. Sebastian, N. F. Zorn, S. Settele, S. Lindenthal, F. J. Berger, C. Bendel, H. Li, B. S. Flavel and J. Zaumseil, *J. Phys. Chem. Lett.*, 2022, **13**, 3542–3548.

12 N. Jiang, *Rep. Prog. Phys.*, 2015, **79**, 016501.

13 J. W. Jordan, K. L. Y. Fung, S. T. Skowron, C. S. Allen, J. Biskupek, G. N. Newton, U. Kaiser and A. N. Khlobystov, *Chem. Sci.*, 2021, **12**, 7377–7387.

14 A. A. Bolzan, C. Fong, B. J. Kennedy and C. J. Howard, *Acta Cryst B*, 1997, **53**, 373–380.

15 D. Böhm, M. Beetz, M. Schuster, K. Peters, A. G. Hufnagel, M. Döblinger, B. Böller, T. Bein and D. Fattakhova-Rohlfing, *Advanced Functional Materials*, 2020, **30**, 1906670.

16 T. Cui, X. Pan, J. Dong, S. Miao, D. Miao and X. Bao, *Nano Research*, 2018, **11**, 3132–3144.

17 J. Wu, Y. Xue, X. Yan, W. Yan, Q. Cheng and Y. Xie, *Nano Research*, 2012, **5**, 521–530.

18 J. F. Moulder, *Handbook of X-ray Photoelectron Spectroscopy: A Reference Book of Standard Spectra for Identification and Interpretation of XPS Data*, Physical Electronics Division, Perkin-Elmer Corporation, 1992.

19 X. Li, Z. Yang, W. Qi, Y. Li, Y. Wu, S. Zhou, S. Huang, J. Wei, H. Li and P. Yao, *Applied Surface Science*, 2016, **363**, 381–388.

20 Q. Huang, J. Zhang, Z. He, P. Shi, X. Qin and W. Yao, *Chemical Engineering Journal*, 2017, **313**, 1088–1098.

21 D. J. Morgan, *Surface and Interface Analysis*, 2015, **47**, 1072–1079.
